# Dendritic fibromyxolipoma of the male breast: case report and literary review

**DOI:** 10.3389/fmed.2025.1653754

**Published:** 2025-12-12

**Authors:** Xiaolan Li, Demian Wu, Xiaoxue Tian, Ting Xu, Qiyao Ge, Shuai Luo, Jinjing Wang

**Affiliations:** 1 Department of Pathology, Affiliated Hospital of Zunyi Medical University, Zunyi, Guizhou, China; 2 Clinical Medicine, Guizhou Medical University, Guiyang, Guizhou, China

**Keywords:** dendritic fibromyxolipoma, differential diagnosis, mammary, pathological characteristics, review

## Abstract

**Background:**

Dendritic fibromyxolipoma (DFML) represents a benign lipomatous tumor. Its rarity and distinctive morphology can lead to misinterpretation of other mesenchymal malignancies, which may result in unnecessary aggressive treatments.

**Case presentation:**

A 39-year-old male presented with a left mammary mass that had been noticed 1 year earlier. Chest computed tomography identified a nodule characterized by fat density in the left mammary region. The initial clinical impression was gynecomastia. A local excision of the left mammary mass was subsequently performed. Histopathological analysis confirmed the diagnosis of DFML. Post-surgical follow-up over 38 months showed no recurrence.

**Conclusion:**

DFML is a benign lipomatous tumor with unique morphological features. Complete surgical excision typically leads to no recurrence. This report documents the first known instance of DFML in the mammary gland of a male patient. Furthermore, an analysis of 95 previously documented cases of DFML was conducted. It is strongly advocated that the World Health Organization reclassify DFML as a mucinous subtype of spindle cell lipoma. This reclassification would improve the understanding of DFML and help avoid unnecessary aggressive treatments due to misdiagnosis as a malignancy.

## Background

Dendritic fibromyxolipoma (DFML) is an uncommon and distinct benign lipomatous neoplasm. Initially reported by Suster et al. (1), fewer than 100 cases have been documented thus far. Nevertheless, this tumor is not yet included in the World Health Organization (WHO) Classification of Soft Tissue and Bone Tumors (2). The cytogenetic features of DFML align with those of spindle cell lipomas (SCL), indicating that DFML might be categorized as a mucinous subtype of SCL. It is advocated that the WHO recognize DFML as a specific subtype of SCL to enhance the definition and understanding of this tumor.

## Case demonstration

A 39-year-old male reported a left mammary mass first observed 1 year earlier. Initially, the mass measured about 1 cm, without accompanying redness, swelling, or pain, and no nipple discharge or bleeding was noted. The patient denied experiencing chills, fever, fatigue, appetite loss, low-grade fever, night sweats, chest tightness, chest pain, or respiratory difficulties. Over the year, the mass enlarged to 4 cm. The patient sought further evaluation and management at our institution. Throughout the symptom duration, the patient’s mental state, appetite, and sleep patterns remained unchanged, with normal bowel and urinary functions. On admission, the physical examination documented the following vital signs: temperature 36.5 °C, pulse 72 beats/min, respiratory rate 18 breaths/min, and blood pressure 135/84 mmHg. A detailed examination of the mammary area revealed multiple nodules in both mammary glands. In the left mammary gland, at approximately the 8 o’clock position, a 48 mm× 30 mm× 13 mm mass with well-defined margins was detected. The right mammary gland had a larger nodule measuring approximately 12 mm× 5 mm, located at the 12 o’clock position, approximately 30 mm from the nipple. Multiple well-defined nodules were also palpated in both axillary regions.

After admission, bilateral breast ultrasound revealed the following findings: No significant glandular-like echoes were detected posterior to the bilateral nipples. A mixed-echo mass measuring approximately 48 mm × 30 mm × 13 mm was identified in the left chest wall, near the nipple at about the 8 o’clock position within the muscular layer. The mass exhibited clear boundaries but poor sound transmission in its liquid portion. Punctate low-echo areas were noted within the mass, and no significant internal blood flow signals were detected; however, punctate blood flow signals were observed peripherally. The largest nodule on the right side measured approximately 13 mm × 4 mm, while the largest on the left side was approximately 13 mm × 6 mm. These nodules displayed punctate and linear blood flow signals. Multiple solid hypoechoic nodules with clear boundaries, flat morphology, and distinct cortical-medullary differentiation were detected in both axillary regions. The mixed-echo mass in the left chest wall warranted further investigation based on the ultrasound findings. Additional imaging studies were recommended for a more comprehensive evaluation.

**Figure 1:**
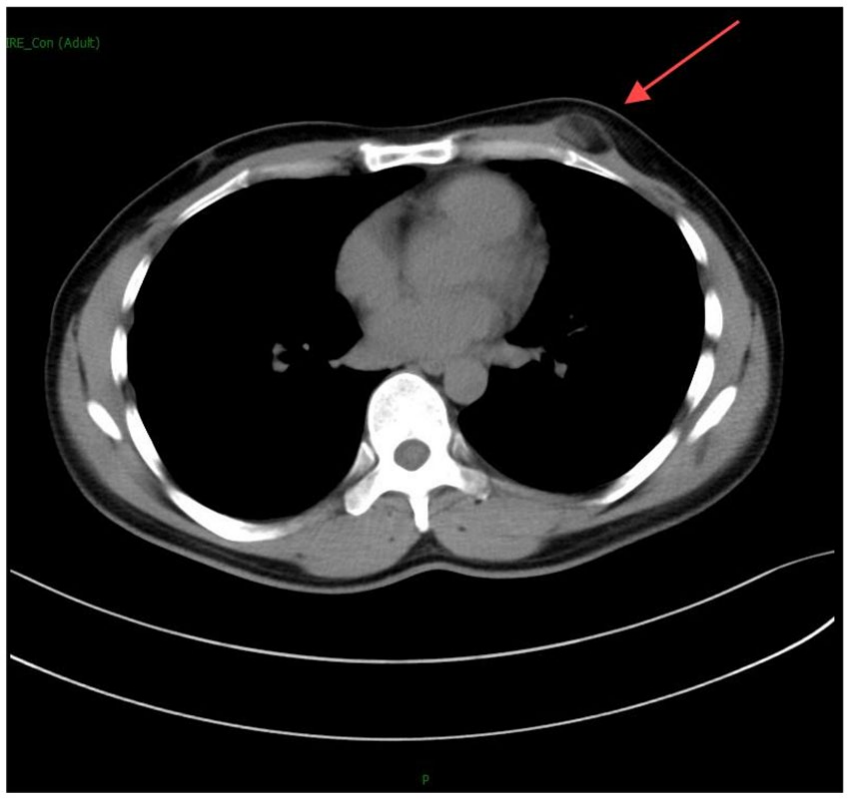
Chest CT scan showing a subcutaneous nodule measuring approximately 15 mm × 25 mm with fat density in the left chest wall. The nodule’s margins appear slightly blurred.

**Figure 2:**
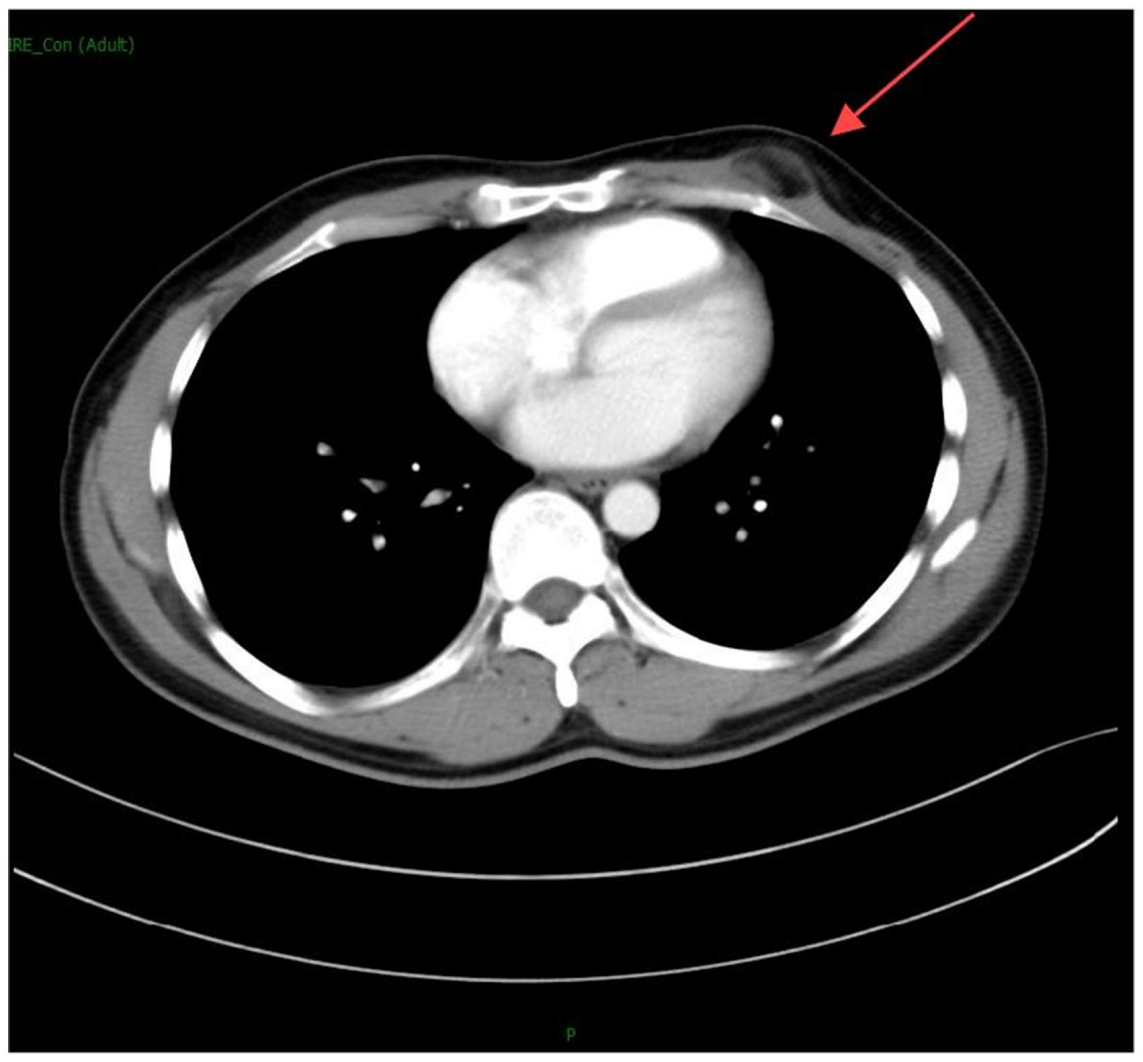
Contrast-enhanced chest CT scan showing no significant enhancement of the left chest wall nodule.

The subsequent chest computed tomography (CT) (Figures 1, 2) revealed the following findings: A subcutaneous nodule measuring approximately 15 mm × 25 mm with fat density was observed in the left chest wall. The nodule exhibited slightly blurred margins and showed no significant enhancement on contrast-enhanced scanning. Small nodular foci and cord-like density increases were noted in both lungs, predominantly in the upper lobes, without abnormal enhancement on contrast-enhanced scanning. Partial bronchiectasis was observed in the left upper lobe, and multiple areas of decreased density were seen in both lungs. The trachea and lobar bronchi were patent. The mediastinum was centrally positioned, and no enlarged lymph nodes were detected in the mediastinum or bilateral hilar regions. The heart size and morphology appeared normal. A peripherally enhancing nodule was observed in the right lobe of the liver within the scanned area. Moreover, CT findings suggested the following diagnoses: (1) A subcutaneous fat-containing nodule in the left chest wall, consistent with lipoma or angiolipoma; (2) Secondary pulmonary tuberculosis in both lungs, primarily characterized by fibroproliferative foci, with partial bronchiectasis in the left upper lobe and multiple areas of emphysema in both lungs; and (3) A lesion in the right lobe of the liver, suspected to be a hemangioma. Additionally, chest radiography, electrocardiogram, complete blood count, electrolytes, liver and kidney function tests, coagulation profile, and hepatitis B panel showed no significant abnormalities. Tests for HIV, syphilis, and hepatitis C were all negative.

After the patient’s admission and completion of pertinent examinations, clinical consideration initially leaned towards gynecomastia. Consequently, an excision of the left mammary mass coupled with fascia flap reconstruction was undertaken. Intraoperatively, a solitary mass was identified at the 8 o’clock position within the left mammary gland. The mass, approximately 5 cm × 3 cm in size, exhibited an intact capsule, soft texture, clear boundaries, and was mobile. It was meticulously separated and completely excised, along with some adjacent glandular tissue.

Postoperatively, the excised tumor mass was submitted for histopathological examination. Gross examination revealed an oval-shaped mass with distinct boundaries, measuring 47 mm × 32 mm × 13 mm. The mass displayed a grayish-yellow to grayish-white coloration. The cut surface exhibited a solid consistency, varying from soft to medium in texture, with a gelatinous or myxoid appearance.

**Figure 3:**
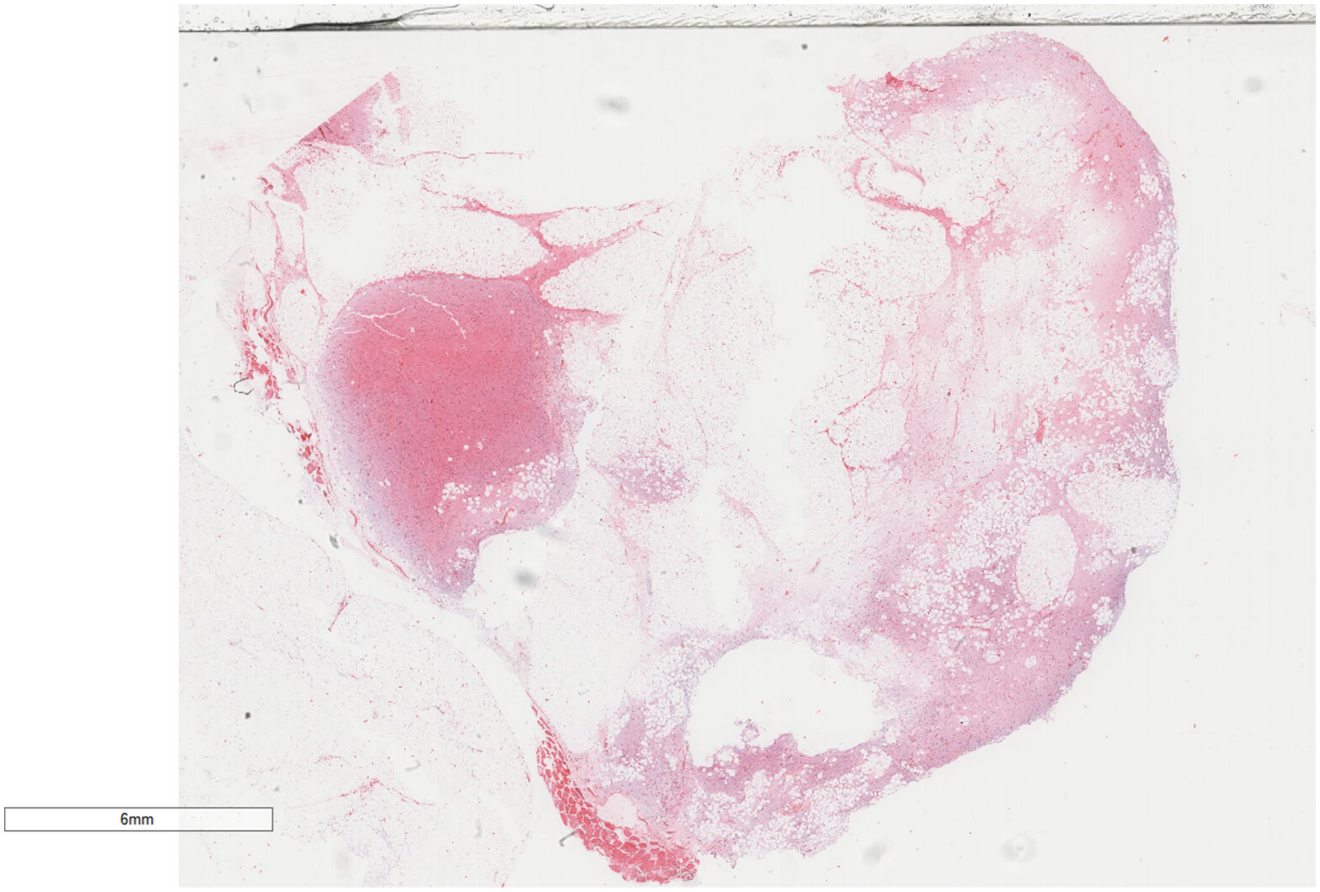
Under low magnification, small spindle cells, stellate cells, and mature adipocytes are observed within a myxoid stroma and a cord-like (scar-like) collagen fiber background. H&E × 4.

**Figure 4:**
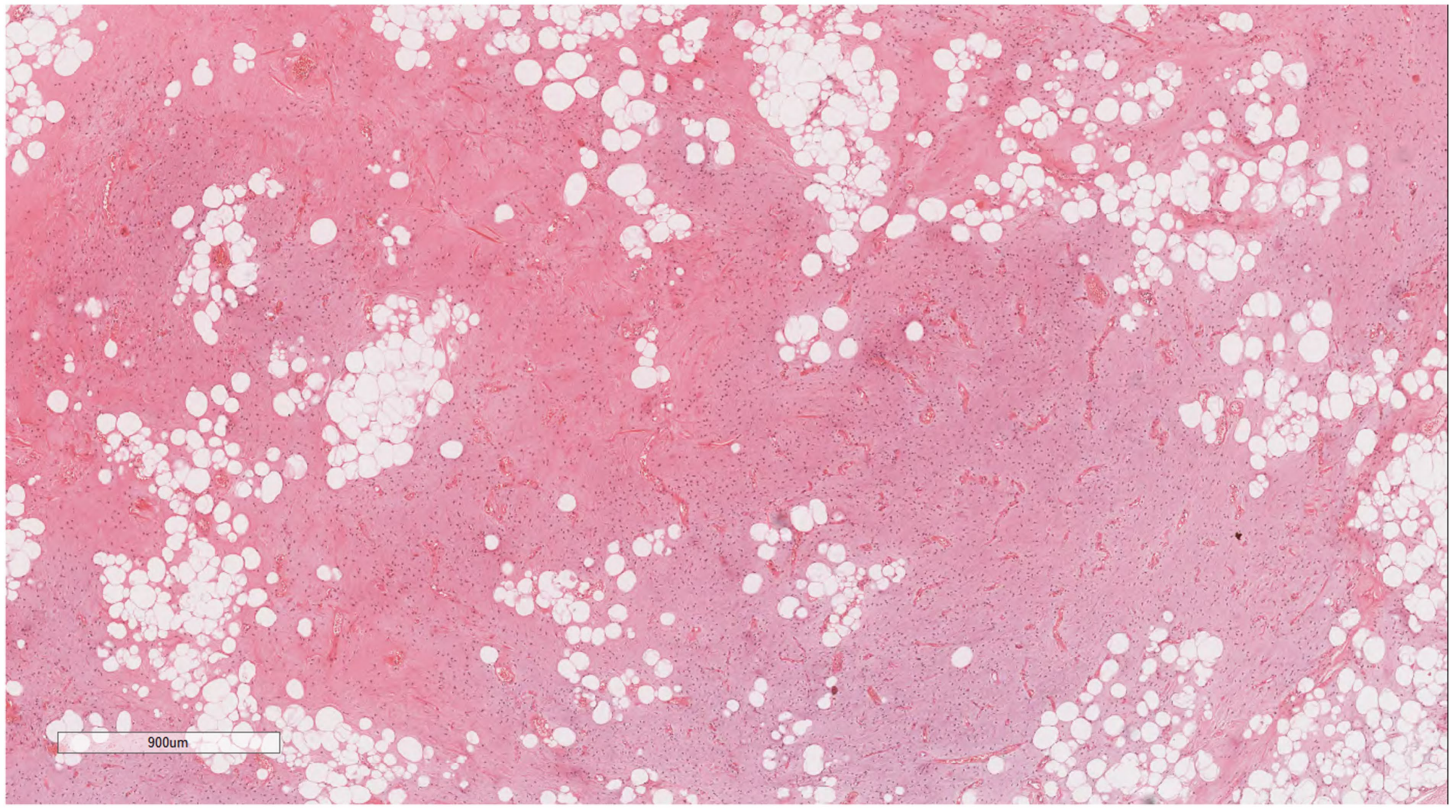
Microscopic examination reveals numerous thin-walled small blood vessels in the tumor stroma, occasionally appearing in clusters. H&E × 20.4.

**Figure 5:**
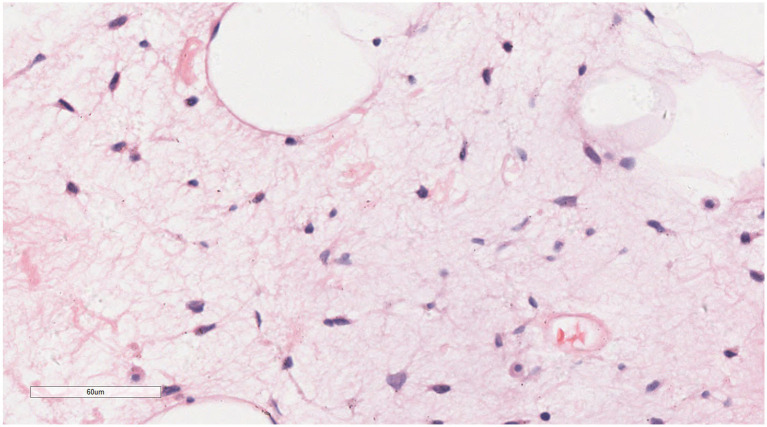
High magnification reveals spindle and stellate cells with small, darkly stained nuclei without atypia or mitotic figures. H&E × 400.

**Figure 6:**
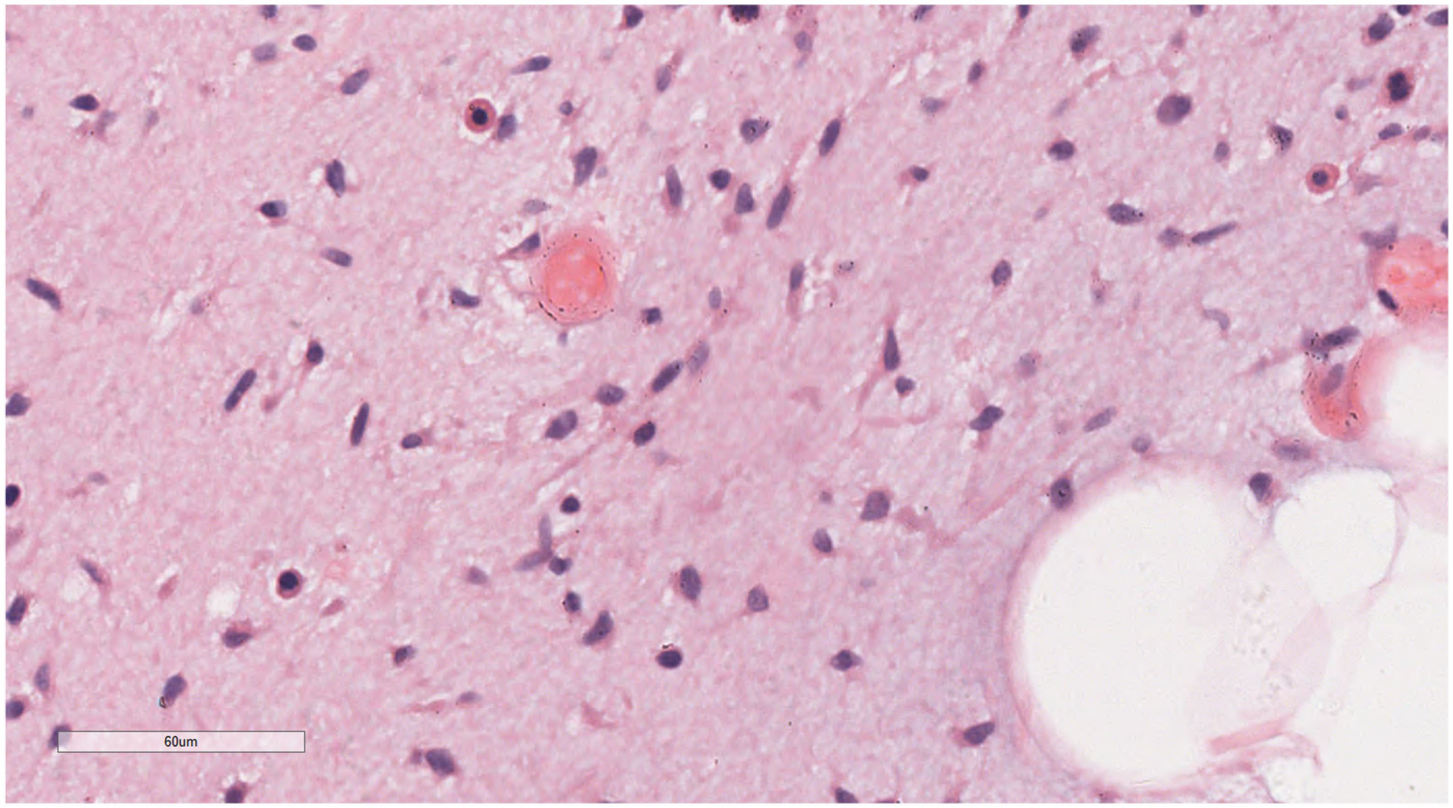
Characteristic stellate-shaped tumor cells with slender dendritic-like cytoplasm are observed within the myxoid stroma. H&E × 400.

Under low magnification (Figures 3, 4), a mixture of small spindle cells, stellate cells, and mature adipocytes in varying proportions was noted against a background of myxoid stroma and cord-like (scar-like) collagen fibers. The adipocytes were fully differentiated, with no presence of immature lipoblasts or adipocytes at other stages of differentiation. The tumor stroma contained numerous thin-walled small blood vessels, occasionally forming clusters, and scattered mast cells. Under high magnification (Figures 5, 6), the spindle and stellate cells exhibited small, hyperchromatic nuclei without signs of atypia or mitotic activity. Characteristic stellate-shaped tumor cells with long, dendritic cytoplasmic processes were distributed within the myxoid stroma.

**Figure 7:**
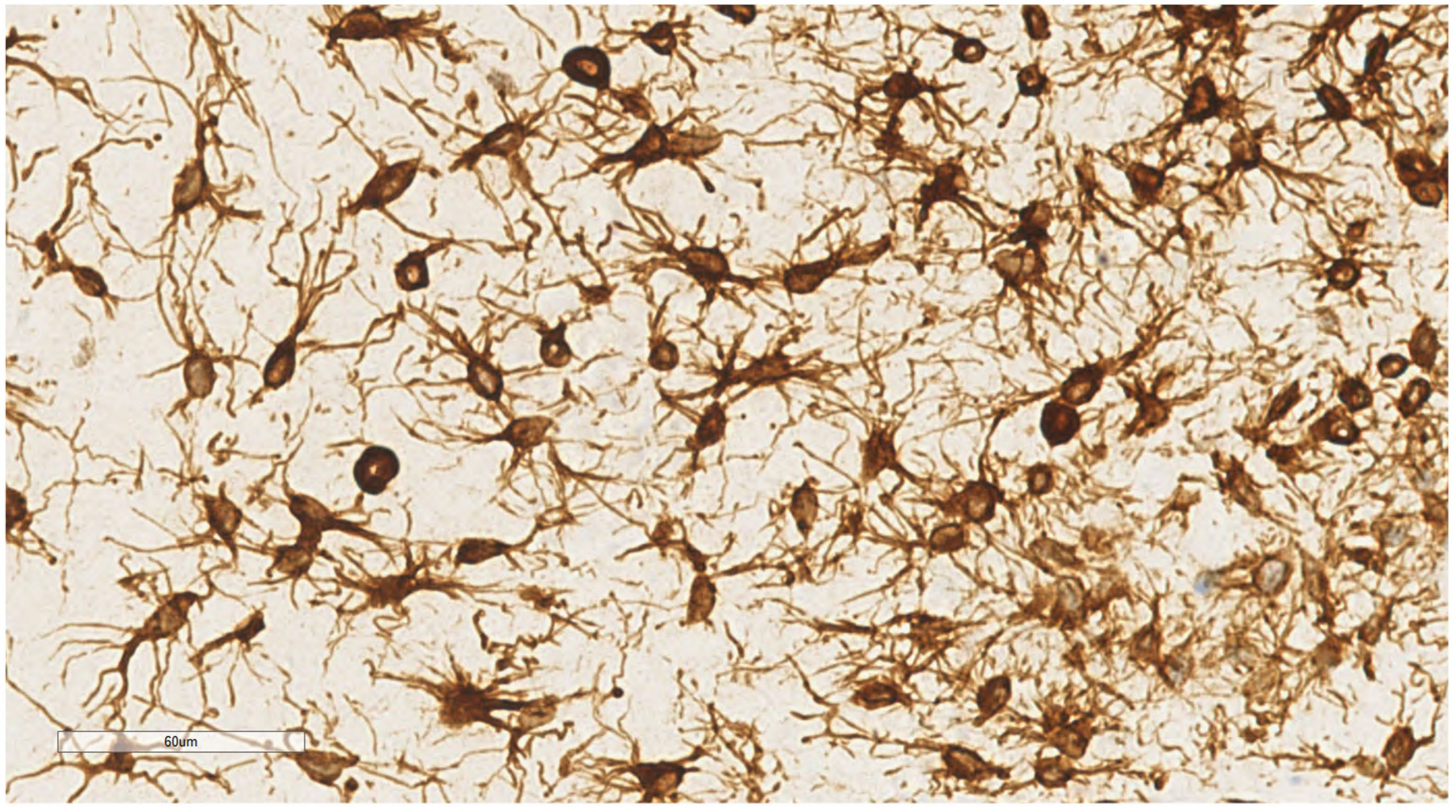
Stellate and dendritic cells show strong positive staining for vimentin, outlining the contours of the dendritic cells. EnVision ×400.

**Figure 8:**
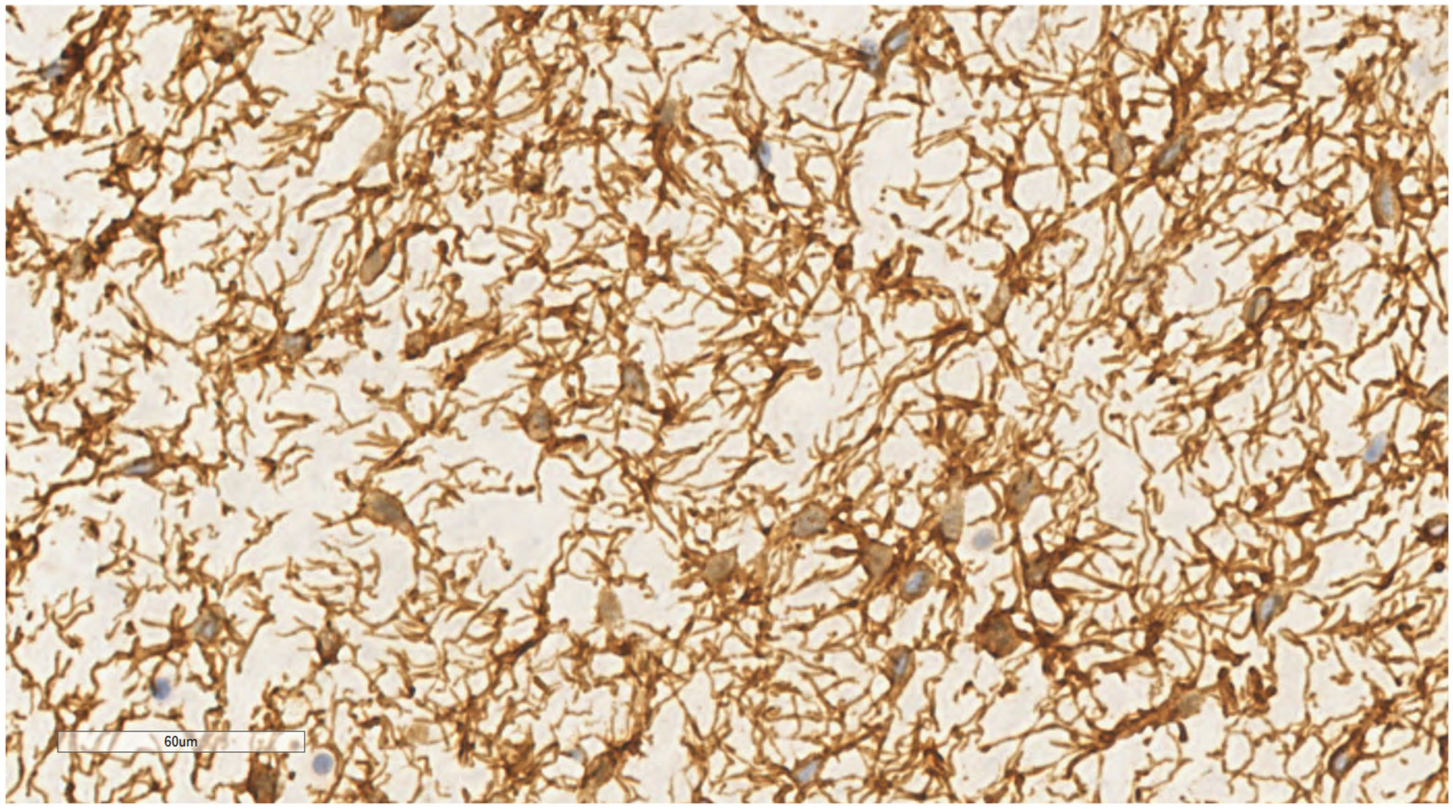
Stellate and dendritic cells show strong positive staining for CD34, highlighting the contours of the dendritic cells. EnVision ×400.

Immunohistochemical analysis showed that both spindle cells and stellate cells were strongly positive for vimentin (Figure 7) and CD34 (Figure 8), displaying prominent dendritic cytoplasmic processes. BCL-2 showed diffuse/focal positivity. Some mature adipocytes were positive for S-100, while scattered mast cells were positive for CD117. The Ki-67 proliferation index was consistently < 1%. Negative results were obtained for CK, desmin, SMA, HMB-45, MDM2, CDK4, p16, β-catenin, and STAT6.

**Figure 9:**
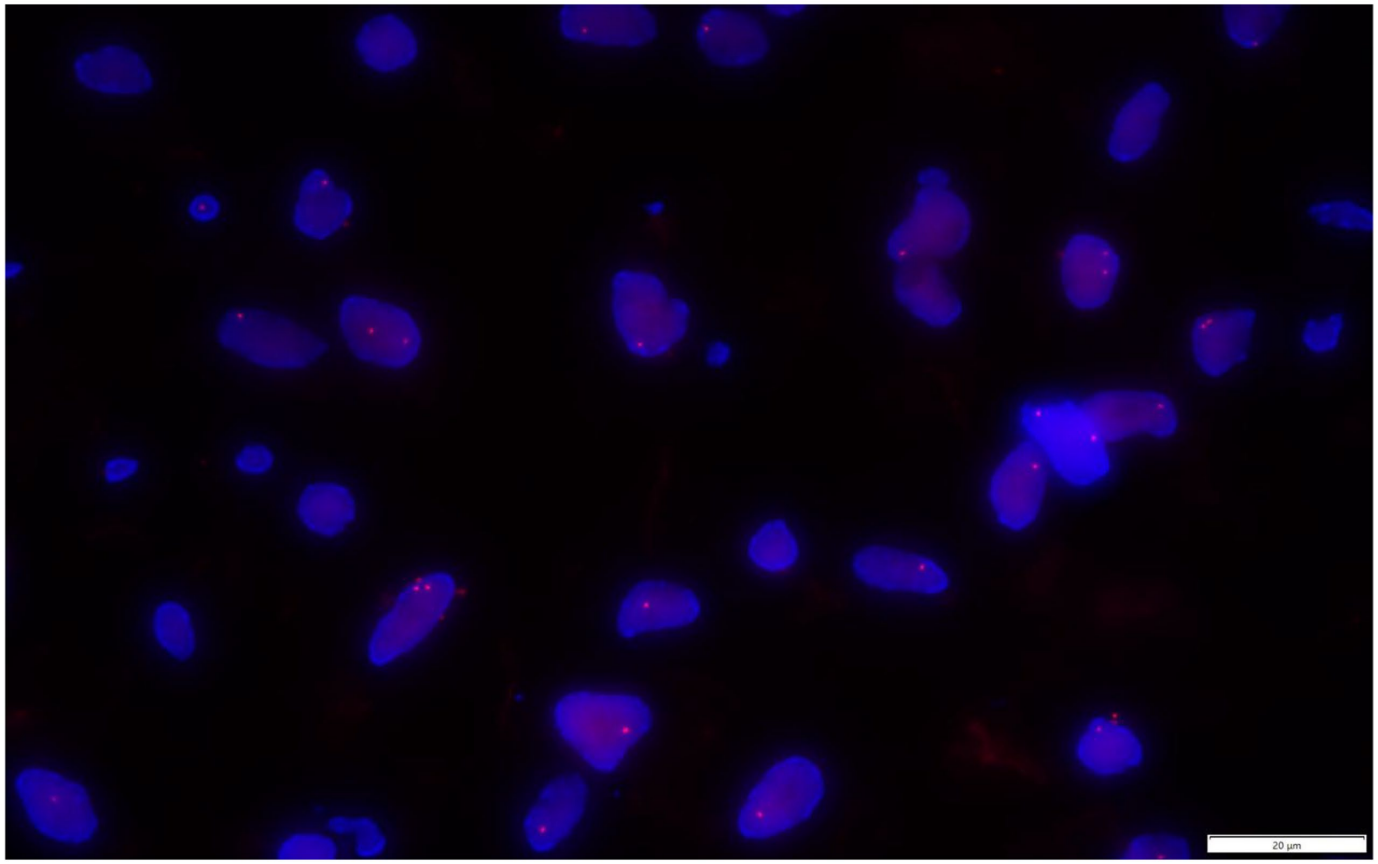
FISH analysis reveals a deletion in the 13q14region of the tumor (a nucleus with 2 red signals is no deletion, and a nucleus with 1 red signal is deletion).

Molecular testing (Figure 9) via fluorescence *in situ* hybridization (FISH) analysis identified a deletion in the 13q14region of the tumor, indicative of RB1 gene loss.100 tumor cells were counted, 2 red signals 18, 1 red signal 82. RB1(13q14) gene deletion ratio:82%, (Note: deletion ratio greater than the threshold of 20% is defined as positive, indicating deletion).

Considering the clinical history, histomorphological features, immunohistochemical findings, and molecular testing results, the diagnosis is (left mammary) DFML.

Postoperatively, the patient’s overall condition remained satisfactory. A 38-month follow-up period showed no recurrence.

## Discussion

The search results from PubMed, CNKI, and Wanfang databases were analyzed using the search terms “dendritic fibromyxolipoma” or “DFML.” The inclusion criteria were case reports or case series of patients with DFML. The exclusion criteria were commentaries, news articles, letters, and articles that did not meet the inclusion criteria. Between 1998 and June 16, 2021, a total of 39 articles were identified. Among these, 18 English-language articles reported 31 cases, while 21 Chinese-language articles reported 64 cases. Collectively, 95 cases of DFML have been reported (Table 1).

**Table 1:** A literature review of 95 DFML cases.

Time	Author	Number of cases	Sex	Age	Diameter/cm	Position	Recurrent condition
1998	Suster et al. (1)	12	M	33	11	Back shoulder	No
			M	54	5	Right nape	No
			M	58	7.5	Right shoulder	No
			M	63	6	Upper back	No
			M	66	8	nape	No
			M	66	9	The axillary wall of the back	No
			M	70	2	Right nose	No
			M	73	7	Right nape	No
			M	77	3	nape	No
			M	79	3.5	Right chest wall	No
			M	81	3.5	Left chest wall	No
			F	50	6	Right upper back	No
2003	Karim et al. (3)	1	M	73	13	Right shoulder, between the subcutaneous muscle and the deltoid	No
2003(CNKI)	Tang (4)	8	M	74	5.5	Left leg	No
			M	75	3.9	nape	No
			M	45	3	Lower back	No
			M	60	2	Left toe	No
			M	67	2.8	Right neck	No
			M	74	5	Right shoulder	No
			M	56	2	Left superior orbit	No
			M	70	9.5	Right leg	No
2009(CNKI)	Chao et al. (5)	1	M	44	5	Dorsum of right foot	No
2011	Al-Maskery et al. (6)	1	F	36	2	Labium inferior	No
2011(CNKI)	Jin et al. (7)	1	M	40	4	Left parotid gland	Recurrence after 1 year
2012	Dahlin and Ljungberg (8)	1	F	65	3.2	Left forearm, attached to the median nerve	No
2012(CNKI)	Qiao et al. (9)	10	M	52	1	neck	No
			M	59	3.5	Left clavicle	No
			M	39	2.5	Left buttock	No
			M	43	2.5	Right parotid gland	No
			F	57	6	Right shoulder	No
			M	63	3.5	Left back	No
			M	54	3	Right neck	No
			M	51	4.5	Right anterior axillary	No
			M	54	5.5	Right back	No
			M	67	4	Left nape	No
2013(CNKI)	Zhang et al. (10)	1	F	32	20	Right groin	No
2014	Han et al. (11)	1	M	69	1	Tip of the nose	No
2014	Wong et al. (12)	1	M	67	7	Left shoulder	No
2014(CNKI)	Geng et al. (13)	1	F	46	5.5	Left upper arm	No
2014(CNKI)	Yuan et al. (14)	3	M	68	5.9	Left forearm (intermuscular)	No
			M	64	8.6	Left forearm (intermuscular)	No
			M	50	3.7	Occipital part	No
2015	Liu et al. (15)	1	M	53	2	Right dorsi (intramuscular lats)	No
2015	Xu et al. (16)	1	M	24	14	Left shoulder, triceps (intramuscular)	No
2015(CNKI)	Guo et al. (17)	5	M	45	5	neck	No
			M	51	3	Wall of the chest	No
			M	58	6	back	No
			M	63	7.5	Regio scapularis	No
			M	80	8	neck	No
2016	AlAbdulsalam and Arafah (18)	1	M	38	3.4	Pyriform recess	No
2016	Ciloglu et al. (19)	1	F	59	17	Left groin area	No
2016(CNKI)	Xia et al. (20)	2	M	72	8	Shoulder and back	No
			F	68	5	Left thigh	No
2016(CNKI)	Song et al. (21)	1	F	34	4	submaxillary	Recurrences 10 years after resection
2016(CNKI)	Guo and Zhang (22)	1	F	2	6	Left thigh	No
2017(CNKI)	Xiao et al. (23)	2	M	38	10	Left abdominal cavity	No
			F	53	7	Peritoneal cavity	No
2017(CNKI)	Zhu et al. (24)	1	M	52	9.1	Right axilla	No
2017(CNKI)	Li et al. (25)	6	M	73	10	Right shoulder	No
			M	40	6	Regiones dorsales nuchae	No
			M	48	2.5	Right thigh	No
			M	47	18.5	mesentery	No
			F	67	9	Right iliac fossa	No
			F	27	10	Left lumbar dorsum	No
2018	Ruiz et al. (26)	1	M	69	5	Subclavian region	No
2018(CNKI)	Fu et al. (27)	3	M	48	5.9	Right neck	No
			M	62	4.3	Cavum oropharyngeum	No
			F	41	5.5	Right shoulder	No
2018(CNKI)	Ding et al. (28)	1	F	56	3.5	Gastric submucosa	No
2019	Tang et al. (29)	1	F	24	2.1	Parotid gland	No
2019(CNKI)	She et al. (30)	3	M	36	3	neck	No
			F	24	3	Parotid gland	No
			M	51	5	neck	No
2019(CNKI)	Li et al. (31)	5	M	50	6	hip	No
			M	50	6	Partes iliaca	No
			M	50	6	Anterior tibia	No
			M	50	6	Plantaris pedis	No
			F	50	6	thigh	No
2019(CNKI)	Sun and Wang(32)	1	M	57	4.3	Right upper arm	No
2020	Jin and Zhang (33)	1	F	6	5.3	back	No
2021	Liu et al. (34)	3	M	50	7.9	Right upper arm	No
			M	33	3	Right thigh	No
			M	48	6	Left mediastinum	No
2021(CNKI)	Song et al. (35)	2	M	39	2	Subcutanea temporo-occipitalis left	No
			M	31	3	Wall of the chest	No
2022	Ji et al. (36)	1	M	55	5.6	Right thigh	No
2022(CNKI)	Luo et al. (37)	5	M	6	7.5	Right axilla	No
			F	35	1	Right arm	No
			F	51	3	Right thigh	No
			M	57	2	Left leg	No
			M	51	2.5	Occipital scalp	No
2023	Li et al. (38)	1	F	11	12.5	Right thorax	No
2023	Tang et al. (39)	1	F	70	2	cardia	No
2024	AlSalman et al. (40)	1	M	38	4	Right shoulder	No
2024	Our case	1	M	39	4	Left mammary gland	No

DFML is a rare benign lipogenic tumor. A statistical analysis was conducted on 95 cases of DFML presented in Table 1, providing the most comprehensive and systematic analysis and summary to date of its clinical presentation characteristics, pathological features, immunohistochemistry, electron microscopy findings, and molecular genetic characteristics.

### Clinical features

#### Age and gender

DFML predominantly impacts middle-aged and elderly males, with onset ages ranging from 2 to 81 years. The mean and median age of onset is 52 years. Female patients generally develop the condition at a younger age than males (median age of 46 years for females and 54 years for males). The male-to-female ratio is 72:23 (3.13:1).

#### Site of occurrence

**Figure 10:**
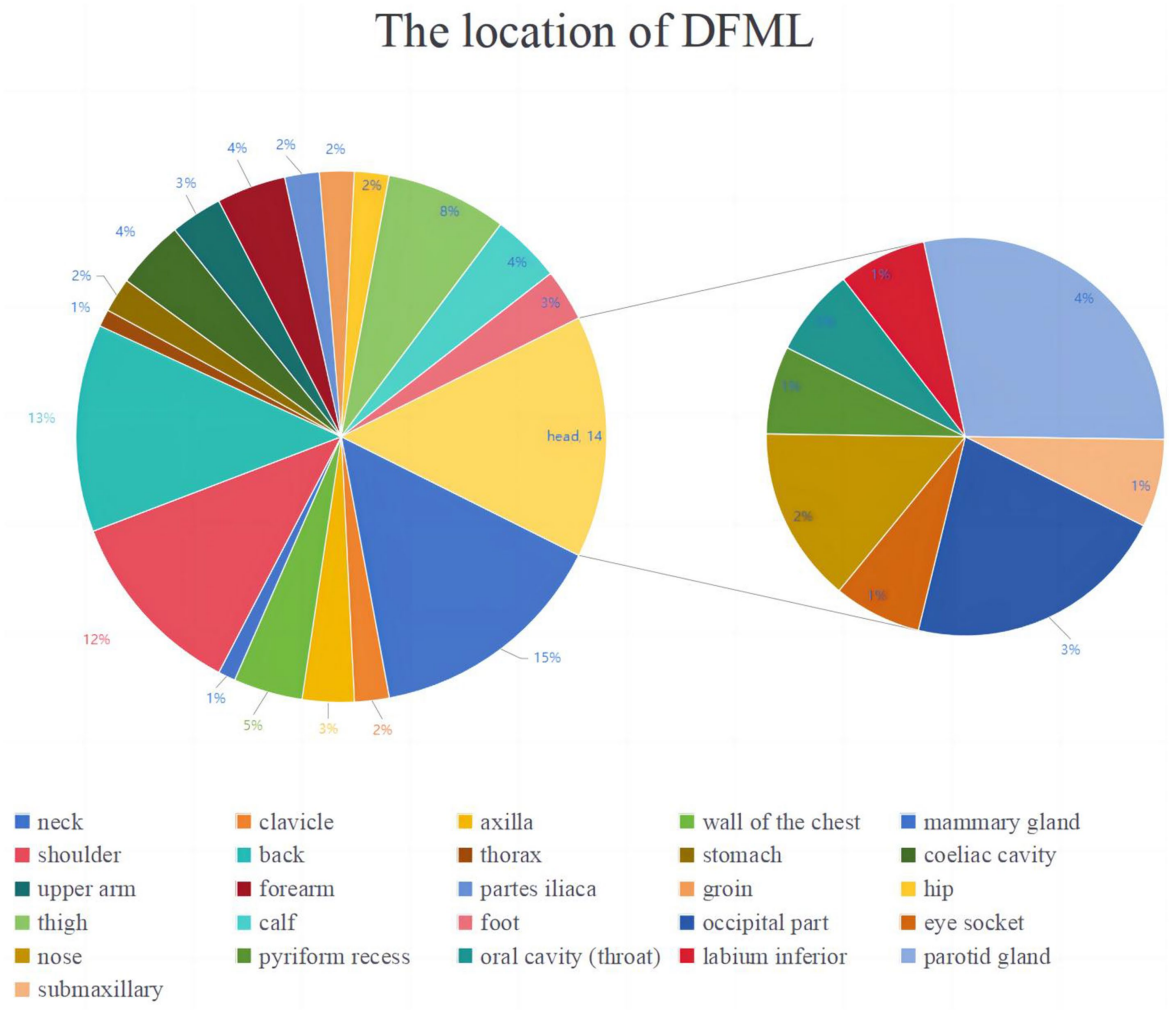
Composite pie chart illustrating the location of DFML.

DFML typically appears in the superficial subcutaneous tissues throughout the body, frequently in the head and neck, shoulders, back, and extremities. Additionally, it can manifest in the chest wall, axilla, and buttocks. It has also been documented in the submucosal layer of the stomach (28, 39), intramuscularly (14, 15), and within the abdominal cavity (23, 25, 41). The case described here, located in the male mammary gland, is the first recorded instance of DFML in mammary tissue Figure 10.

#### Tumor diameter and growth rate

Tumor diameters range from 1 to 20 cm, with an average size of 5.6 cm. The growth pattern can be static, slow-growing, or exhibit rapid enlargement over a short period.

### Pathological characteristics

#### Gross examination

DFML typically manifests with well-defined borders, either partially or completely encapsulated by a thin capsule. The tumor size varies from 1 to 20 cm in diameter, averaging 5.6 cm. Cross-sectional analysis reveals the tumor to be grayish-white to grayish-yellow, with a soft to medium consistency. Most cases display a gelatinous or myxoid appearance, although some may exhibit cystic changes (37).

#### Microscopic features

Microscopic examination of DFML reveals a mix of small spindle cells, stellate cells, and mature adipocytes in varying proportions, set within a myxoid stroma and cord-like (scar-like) collagen fibers. Under high magnification, the spindle and stellate cells present small, hyperchromatic nuclei without atypia or mitotic figures. Notably, the stellate tumor cells feature slender dendritic-like cytoplasmic processes extending into the myxoid stroma. The tumor contains thin-walled small blood vessels, sometimes clustered, and scattered mast cells throughout the stroma (37). In this particular case, dendritic fibers are prominently highlighted under microscopic examination, especially when stained with vimentin and CD34 immunohistochemistry, appearing more pronounced than previously reported.

#### Electron microscope features

Electron microscopy reveals characteristic tumor cells with dendritic cytoplasmic processes resembling fibroblasts. These cells are rich in Golgi apparatus, rough endoplasmic reticulum, mitochondria, and scattered intermediate filaments. Localized pinocytotic activity is observed in the cytoplasmic processes, which lack basement membrane material (1).

#### Immunophenotype

In DFML, spindle and stellate tumor cells exhibit high expression of vimentin and CD34. Staining for vimentin and CD34 highlights the cytoplasmic projections of these characteristic tumor cells, giving them a distinct dendritic appearance. BCL-2 is diffusely or focally positive in both spindle and dendritic cells of DFML (42). CD117 marks scattered mast cells within the stroma, and some mature adipocytes test positive for S-100. Tumor cells do not express epithelial (CK), myogenic (desmin, SMA), neurogenic (CD56, NSE) markers, well-differentiated/dedifferentiated liposarcoma markers (MDM2, CDK4, p16), or solitary fibrous tumor (SFT) markers (β-catenin, STAT6). The Ki-67 proliferation index remains below 1%. In this case, spindle and stellate tumor cells were strongly positive for vimentin and CD34, and negative for CK, desmin, SMA, HMB-45, MDM2, CDK4, p16, β-catenin, and STAT6.

#### Molecular genetics

Among the 95 reported cases of DFML, only one study performed FISH analysis on chromosome 13q14.3, which revealed a deletion of the D13S319 fragment in this region (12). A few studies tested the MDM2 gene and DDIT3 fusion gene to distinguish between liposarcoma and myxoid liposarcoma (MLS), but no deletions or mutations were identified (35). In this case, FISH analysis confirmed a deletion in the 13q14region, indicating the loss of the RB1 gene.

Chromosome 13q14.3 encodes the RB1 gene, and deletions or rearrangements in this region are commonly observed in SCL. Additionally, such genetic alterations are seen in atypical spindle cell/pleomorphic lipomatous tumors, cellular angiofibroma, mammary-type myofibroblastoma, and digital fibromyxoma (43).

Given that DFML and SCL share identical molecular genetic characteristics (13q14.3 deletion), along with similar clinical, pathological, and immunophenotypic features, it has been proposed that DFML is essentially a mucinous subtype of SCL (9). Consequently, a strong recommendation has been reiterated for the next edition of the WHO classification to categorize DFML as a mucinous subtype of SCL.

### Differential diagnosis

The morphology of DFML is distinct and rare, with pathological features that resemble various benign and malignant soft tissue tumors, leading to potential misdiagnosis in clinical pathology. Differentiation from the following tumors is essential:

(1) SCL: DFML and SCL are highly similar. However, SCL lacks significant myxoid stroma and characteristic dendritic cytoplasmic projections (44). Despite this, DFML can be regarded as a spindle cell variant subtype of SCL.
(2) MLS: DFML does not contain the diagnostic lipoblasts found in MLS. MLS cells are S-100 positive and CD34 negative, whereas DFML is strongly CD34 positive. Additionally, over 90% of MLS cases exhibit DDIT3 fusion genes when tested by FISH (45).
(3) Myxoma: The fibrous components and myxoid stroma of myxoma are nearly identical to those of DFML. However, myxoma contains almost no mature adipocytes, and CD34 expression in myxoma is generally only focally positive.
(4) Myxoid SFT: Tumor cells in SFT are arranged alternately in cell-rich and cell-sparse areas, with some regions displaying typical hemangiopericytoma-like changes. Mature adipocytes are nearly absent in the tumor. Immunophenotypically, SFT is positive for STAT6, *β*-catenin (11), and CD99. If necessary, molecular detection of the NAB2-STAT6 fusion gene (46), which is relatively specific to SFT, can be performed for differential diagnosis.

In this case, the diagnosis of DFML is confirmed by integrating histopathological features, immunohistochemical results, and molecular testing findings.

### Treatment and prognosis

DFML exhibits favorable biological behavior, and local excision of the mass is generally curative. Among the 95 cases reported in the literature, only 2 instances of recurrence were documented, likely due to incomplete surgical resection (7, 21). In the present case, the patient’s overall condition was satisfactory post-surgery, with no recurrence observed during a 38-month follow-up period.

## Conclusion

DFML is a rare and morphologically distinctive benign lipoma that primarily affects the superficial soft tissues of middle-aged and elderly males. Clinically, complete excision of the tumor typically suffices for treatment, with no recurrence or metastasis generally observed. Due to its rarity and unique morphological characteristics, DFML can easily be mistaken for various tumors with similar morphology. Therefore, it is strongly recommended that the WHO classify DFML as a mucinous subtype of SCL. This reclassification would improve the understanding of DFML and prevent potential overtreatment resulting from misdiagnosis as a malignant tumor.

## Data Availability

The original contributions presented in the study are included in the article/supplementary material, further inquiries can be directed to the corresponding author.
